# Epidemiology of undiagnosed type 2 diabetes mellitus among hill tribe adults in Thailand

**DOI:** 10.1038/s41598-022-07977-9

**Published:** 2022-03-10

**Authors:** Tawatchai Apidechkul, Chalitar Chomchoei, Panupong Upala

**Affiliations:** 1 Center of Excellence for The Hill Tribe Health Research, Mae Fah Luang University, Chiang Rai, Thailand; 2 School of Health Science, Mae Fah Luang University, Chiang Rai, Thailand

**Keywords:** Epidemiology, Diseases, Medical research

## Abstract

A community-based cross-sectional study was performed to estimate the prevalence of and determine factors associated with undiagnosed type 2 diabetes (T2DM) among hill tribe adults aged 30 years and over. Data were collected from the target hill tribe people living in the 30 selected villages in Chiang Rai Province in northern Thailand. A total of 2365 people were invited to participate in the study; 65.9% were female, 72.4% were aged 40–69 years, 0.9% regularly received preventive healthcare, and only 41.2% understood Thai. The overall prevalence of undiagnosed T2DM was 7.5%. After adjusting for age and sex, two factors were found to be associated with T2DM: triglycerides and high-density lipoprotein cholesterol (HDL-C). Those with high triglyceride levels were 2.80 (95% CI 1.99–3.94) times more likely to be suffering from T2DM than those with a normal triglyceride level. Those with low HDL-C levels were 1.65 (95% CI 1.16–2.34) times more likely to be suffering from T2DM than those with normal HDL-C. Appropriate public health interventions should be developed and implemented to reduce T2DM by providing regular preventive healthcare, educating patients on DM prevention and control, and encouraging regular exercise and reduced consumption of fatty food.

## Introduction

Type 2 diabetes mellitus (T2DM) has been defined as a major noncommunicable disease (NCD), especially in the adult population with limited education and poor economic statuses^1^. A large amount of money has been expended for its treatment and care globally^2^. Due to its complex pathogeneses, T2DM is recognized as one of the most significant threats to individual health and national health systems in both developed and developing countries, including Thailand, particularly among people aged 30 years and over^3–5^. In 2019, Thailand was estimated to have 4.8 million adults (45.8 million people aged 25 years and over) living with T2DM, and this figure is projected to increase to 5.3 million by 2039^6^. Among those with T2DM, only 35.6% have been diagnosed and treated properly, and the disease causes approximately 200 deaths per day^7^.

Undiagnosed T2DM could lead to severe complications in later years and increase medical expense^7^. Moreover, undiagnosed T2DM patients will face a reduced quality of life^8^. Complications from T2DM tend to increase among people living with limited education and poor economic statuses^9^. While several risk factors for T2DM have been identified across various populations^7^, including marital status^10^, elevated triglycerides^11^, and knowledge about DM prevention and control^12^, the exact factors related to undiagnosed T2DM in people living in a certain community have not been clearly identified, especially among the highly vulnerable population. Epidemiological data from Thailand indicate that the population vulnerable to T2DM comprises those aged 30 years and over, and those with a low education level and who live in poor economic conditions, such as hill tribe people, are at higher risk^13^.

Hill tribes are composed of people who migrated from southern China to Thailand over two centuries, and approximately 4 million hill tribe people lived in Thailand in 2019^14^. A large proportion of the hill tribe people settled along the Thailand–Myanmar–Laos borders, far from any cities^15^. Hill tribe farms with traditional agricultural methods grow uneconomic crops such as corn, rice, and beans^16^, which account for 88.1% of the total areas cultivated by these people^17^. Only a small proportion are fluent in the Thai language^17^. Hill tribes have their own lifestyle, culinary traditions, substance use and other health-related behaviors^18^. Almost all of the hill tribe people work in the agricultural sector, growing traditional and noneconomic crops^16^. There is no information available on the factors associated with undiagnosed T2DM in people living in the hill tribe community.

This study aimed to estimate the prevalence of and factors associated with undiagnosed T2DM among hill tribe adult individuals aged 30 years and over in Thailand. The findings can support health policy formation and decision-making in health care services and could also be used to develop proper public health interventions for hill tribe people living in Thailand and neighboring countries.

## Materials

### Study design and study setting

A community-based cross-sectional study design was employed to collect data from the participants and analyze them. The study settings were 10 out of 18 districts in Chiang Rai Province where hill tribe villages are located.

### Study population

The study population was hill tribe adults aged 30 years and over living in Chiang Rai Province. Those eligible to participate belonged to one of six main tribes, Akha, Lahu, Yao, Hmong, Karen, and Lisu, and were living in the study area on the date of data collection. Those who had been previously diagnosed with diabetes, could not provide essential information for the study protocols, and could not identify whether they belonged to one of the six main hill tribes were excluded from the study.

### Study sample

In 2018^15^, there were 749 hill tribe villages in Chiang Rai Province, Thailand: 316 Lahu villages, 243 Akha villages, 63 Yao villages, 56 Hmong villages, 36 Karen villages, and 35 Lisu villages. Roughly 98.0% of the hill tribe people living in these areas belong to either Akha, Lahu, Hmong, Yao, Karan, or Lisu. Five villages of each tribe were randomly selected from the lists of the hill tribe villages. All villagers living in the 30 selected villages who met the eligibility criteria were invited to participate in the study. Three days before the study date, villagers were provided information on the study, including the eligibility criteria.

### Sample size

The sample size was calculated from the standard formula^19^ n = [Z^2^α/_2_ × P × Q]/e^2^, where Z is the value from the standard normal distribution corresponding to the desired confidence level (Z = 1.96 for 95% CI), P = expected true proportion (based on a previous study), and e = the desired precision, which is 0.04. Based on P = 0.18^20^, Q = 0.82, and e = 0.04, at least 354 participants were needed from each tribe, which translated to a total of 2126 participants among the 6 tribes. Adding 10.0% to account for error during the study, at least 2338 participants were required for the analysis.

### Research instruments and their development

A questionnaire was designed specifically for this study, and its validity was tested before it was used in the field. The questionnaire was divided into 4 parts. In part one, nine questions were designed to collect general information of the participants, such as age, sex, and tribe. In part two, nine questions collected information on health-related behaviors such as smoking and alcohol use. In part three, the standard questions of the Stress Test-5 (ST-5)^21,22^ were used to assess the level of stress. In part four, twenty questions assessed the participants’ knowledge and opinions about diabetes prevention and control. In the last part, six blank spaces were provided to record personal health data from the physical examination, such as weight, height, and blood pressure.

In the knowledge section, 10 validated questions assessed the level of knowledge related to the natural history of the disease and the risk factors for diabetes, including prevention and control methods. For each question, two responses were possible: correct or not correct. In the opinion section, 10 validated questions assessed opinions about diabetes prevention and control. Three choices were provided for each item: agree, neutral, and disagree. The sum of knowledge and opinion scores were used to identify the level of knowledge and opinion.

The questionnaire was assessed for validity and reliability before use. The item-objective congruence (IOC) technique was employed, which asked three external experts (an internal medicine expert, a public health expert, and a nutritional expert) to improve the validity of the entire questionnaire. The comments and scores obtained from the experts were incorporated to revise and improve the questionnaire. There were three factors assessed to improve the quality of the questionnaires: the accuracy of the questions, the feasibility of the questions and the sequence of the questions. Finally, the overall Cronbach’s alpha for the opinion section was 0.73.

Pilot tests were conducted twice in 20 hill tribe people (10 males and 10 females) selected for this purpose aged between 30 and 67 years who lived in two hill tribe villages located in Mae Chan District, Chiang Rai Province, Thailand. The purposes of the pilot study were to adjust the content of the questions to fit the context of the hill tribe people in Thailand and to evaluate the feasibility and sequence of the questions. There were more than 11 significant points that had to be improved during the pilot before the questionnaire could be used for general data collection. The accuracy of the questions, the feasibility of the questions and the sequence of the questions were improved during the pilot test. Finally, the overall Cronbach’s alpha for the opinion section was 0.73.

### Variables

Various participant attributes were recorded as independent variables, including age, sex, tribe, education, income, substance use, stress, depression, dietary behaviors and lipid profiles. Many questions were used to gather data on the dependent variables. One of the questions asked the participant to identify his or her tribe, and the responses were Akha, Lahu, Hmong, Yao, Karen, or Lisu. The answer to the question asking who the respondent resided with could alone, with a spouse, with a child, or with a relative. Questions asking about the respondent’s ability to read, understand, speak, and write Thai had three possible responses: no, little, and fluent. The response of “no” meant that the participant was unable to read, speak, understand, or write Thai. “Little” meant that the participant could interact with the interviewer to answer basic questions but needed additional explanation for complicated words or sentences. The response “fluent” meant that the participant could clearly speak, understand, read and write almost all words and sentences in Thai. All variables in the study were specifically defined according to the following operational definition, including the dependent variable (Table 1). Stress was assessed by the standard form, ST-5^21,22^ which consisted of 5 questions and four answer levels for each question: 0, 1, 2, and 3. The maximum score is 15, and the minimum score is 0. Those who had scores of 0–4 were classified as having low stress levels, while scores of 5–9 indicated moderate stress and scores of 9–15 indicated high stress.

**Table 1:** General characteristics of participants.

Characteristics	n	%
Total	2365	100.0
**Sex**		
Male	870	34.1
Female	1558	65.9
**Age (years)**		
30–39	352	14.9
40–49	581	24.6
50–59	602	25.5
60–69	519	21.9
70–79	248	15.9
≥ 80	63	2.7
Mean = 54.1, SD = 13.1, Min = 30, Max = 108		
**Tribe**		
Akha	677	28.6
Lahu	364	15.4
Hmong	349	14.8
Yao	339	14.3
Karen	379	16.0
Lisu	257	10.9
**Education**		
No education	1801	76.2
Primary school	346	14.6
Secondary school and higher	218	9.2
**Occupation**		
Unemployed	584	24.7
Agriculturist	1153	48.8
Employed	581	24.6
Trad and government officer	47	2.0
**Annual income (baht)**		
≤ 50,000	1683	71.2
50,001–100,000	493	20.8
≥ 100,001	189	8.0
Median = 30,000, IQR = 45,000, Min. = 9,600, Max. = 252,000		
**Marital status**		
Single	133	5.6
Married	1892	80.0
Ever married	340	14.4
**Family members (people)**		
≤ 4	1250	52.9
5–8	960	40.6
≥ 9	155	6.6
**Living with**		
Alone	146	6.2
Spouse	1630	68.9
Child	431	18.2
Relatives	158	6.7
**DM history of father**		
No	1351	57.1
Yes	93	3.9
Do not know	921	38.9
**DM history of mother**		
No	1345	56.9
Yes	121	5.1
Do not know	899	38.9
**Annual health check**		
No	2344	99.1
Yes	21	0.9
**Checking DM status in previous year**		
No	2344	99.1
Yes	21	0.9

Body mass index (BMI) was classified into three levels according to the WHO guidelines^21,22^: underweight (≤ 18.5 kg/m^2^), normal weight (18.51–22.99 kg/m^3^), and overweight (≥ 23.00 kg/m^2^). Total cholesterol was classified into two groups: normal (≤ 199 mg/dL) and high (≥ 200 mg/dL)^23^. Low density lipoprotein cholesterol (LDL-C) was classified as normal (< 100 mg/dL) and high (≥ 100 mg/dL)^23^. High density lipoprotein (HDL-C) was classified as low (< 40 mg/dL) and normal (≥ 40) for males, and low (< 50 mg/dL) and normal (≥ 50) for females^23^. Triglycerides were classified as normal (≤ 149 mg/dL) and high (≥ 150 mg/dL)^23^. Those who had never been diagnosed previously and whose HbA1c ≥ 6.5 mg% were defined as undiagnosed T2DM^24^.

The laboratory tests were conducted at the Mae Fah Luang Medical Laboratory Center. All laboratory procedures used standard equipment. The latex enhanced immunoturbidimetric method (RANDOX@) was used to detect the HbA1c level; the assay was certified by the National Glycohemoglobin Standardization Program and standardized to the Diabetes Control and Complication Trial reference. The direct clearance method was used to detect the total cholesterol, HDL-C, and LDL-C levels, while glycerin phosphate oxidase peroxidase was used for triglyceride detection. The equipment was always validated and calibrated before use. One body weight scale was used to weigh the participants throughout the entire project, and the equipment was always calibrated before use.

### Data collection procedures

Permission to access the villages was granted by the District Government Office. Villages were randomly selected from the list of villages of the six main hill tribes. The headmen of the selected villages were contacted to provide information regarding the research project, and care was taken to clearly describe the study objectives and the inclusion and exclusion criteria. Participants who met the inclusion criteria were contacted three days prior to data collection and were informed about the requirement to abstain from food and drink for at least 8 h before the blood specimens were collected. On the date of data collection, participants were provided all essential information regarding the study protocols and their right to withdraw from the project without any further repercussions, including access to medical services. Afterward, participants were asked to voluntarily sign or stamp their fingerprint on an informed consent form before the physical examination, five mL blood specimen collection, and completion of the questionnaire. People who could not speak Thai were supported by a village health volunteer who was one of the hill tribe people and fluent in Thai. Each data collection process lasted 30 min. Data were collected between November 2019 and March 2020.

### Statistical analysis

Questionnaire data were double entered into Excel sheets and checked for missing data before being transferred to SPSS for the data analysis (Version 24, Chicago, IL). Categorical data, such as sex and tribe, are presented in the form of percentages. Continuous data are presented as the mean and standard deviation (SD) for normally distributed data, and the median and the interquartile range (IQR) are presented for data with a skewed distribution. Binary logistic regression was used to identify factors associated with T2DM at the significance level of α = 0.20 in univariable analysis and 0.05 in multivariable analysis.

### Ethics approval

All of the study concepts and the protocol were approved for research on humans by the Mae Fah Luang University Research Ethics Committee on Human Research (No. REH-6100), which were in accordance with the Declaration of Helsinki. All participants received complete information and were asked about their willingness to participate in the study, and we obtained written informed consent before the data collection. For those who were illiterate and could not sign, they were asked to stamp a fingerprint on the document after the essential information relevant to the study objective and protocols, including the health information, was explained to them in their local language with the help of a village health volunteer who spoke both Thai and local languages, which was approved by the Mae Fah Luang University Research Ethics Committee on Human Research. All participants were informed about the laboratory results, including recommendations for further action if required. This process was helped by public health workers who were working in health-promoting hospitals and village health volunteers.

## Results

A total of 2365 participants were recruited for the analysis (Table S1); 65.9% were female, 72.0% were aged 40–69 years (mean = 54.1, SD = 13.1), 54.0% were Buddhist, 80.0% were married, and 52.9% had ≤ 4 family members. A large proportion had no formal education (76.2%). The majority of participants were farmers and unemployed (73.5%) and had a low family income (less than 50,000 baht/family/year), and 31.0% had family debt. Only a small proportion were able to use Thai: 39.0% were able to speak it, and 18.8% were able to read it. One-fourth (26.9%) had no health insurance or were not covered by the universal coverage of Thailand health insurance, and only 21 persons (0.9%) received preventive healthcare regularly (Table 1).

Five hundred and seventy-seven reported smoking (24.4%), and 23.7% used alcohol. A large proportion did not exercise (61.0%), and 18.5% reported moderate-to-high stress. A large proportion (63.6%) had a low level of knowledge regarding diabetes prevention and control, and 76.2% had negative opinions toward diabetes prevention and control. Regarding body weight, 60.1% were overweight, and 7.9% were underweight. As for lipid profiles, 44.2% were classified as high in total cholesterol, 45.5% were classified as low in HDL-C, 69.6% were classified as high in LDL-C, and 41.6% were classified as high in triglycerides (Table 2).

**Table 2:** Factors associated with T2DM among undiagnosed group in univariable and multivariable logistic regressions.

Factors	Univariable analysis			Multivariable analysis		
	OR	95% CI	p-value	OR	95% CI	p-value
**Sex**						
Male	1.00					
Female	1.01	0.73–1.40	0.948			
**Age (years)**						
30–39	1.00					
40–49	0.83	0.50–1.38	0.477			
50–59	1.05	0.65–1.70	0.849			
60–69	0.76	0.45–1.29	0.308			
70–79	1.07	0.60–1.93	0.821			
≥ 80	1.45	0.60–3.47	0.409			
**Tribe**						
Akha	1.00					
Lahu	1.13	0.69–1.85	0.640			
Hmong	0.85	0.50–1.47	0.568			
Yao	1.22	0.74–2.00	0.441			
Karen	1.30	0.81–2.08	0.283			
Lisu	1.58	0.95–2.62	0.076*			
**Religion**						
Buddhist	1.00					
Christian or Muslim	1.09	0.80–1.48	0.584			
**Education**						
No education	1.03	0.61–1.73	0.919			
Primary school	0.57	0.28–1.16	0.122*			
Secondary school and higher	1.00					
**Occupation**						
Unemployed	1.88	0.44–8.00	0.394			
Agriculturist	1.91	0.46–7.98	0.378			
Employed	1.66	0.39–7.11	0.492			
Trad and government officer	1.00					
**Annual income (baht)**						
≤ 50,000	0.93	0.53–1.63	0.801			
50,001–100,000	0.94	0.51–1.76	0.849			
≥ 100,001	1.00					
**Marital status**						
Single	1.00					
Married	1.78	0.77–4.11	0.175*			
Ever married	1.61	0.64–4.03	0.311			
**Family history of DM**						
No	1.00					
Yes	0.65	0.26–1.63	0.355			
Do not know	0.84	0.61–1.16	0.278			
**Smoking**						
No	1.00					
Yes	1.03	0.72–1.46	0.882			
**Alcohol consumption**						
No	1.00					
Yes	1.11	0.78–1.58	0.570			
**Exercise**						
No	1.34	0.77–2.34	0.299			
Sometimes	1.38	0.76–2.50	0.285			
Regularly	1.00					
**Stress (ST-5)**						
Low	1.00					
Moderate	0.66	0.41–1.07	0.095*			
High	1.20	0.51–2.81	0.683			
**BMI**						
Normal weight	1.00					
Underweight	0.84	0.44–1.59	0.586			
Overweight	1.01	0.72–1.41	0.959			
**Total cholesterol**						
Normal	1.00					
High	0.77	0.57–1.06	0.109*			
**HDL-C**						
Normal	1.00			1.00		
Low	1.07	0.78–1.45	0.690	1.65	1.16–2.34	0.006**
**LDL-C**						
Normal	1.00					
High	0.73	0.53–1.01	0.056*			
**Triglycerides**						
Normal	1.00			1.00		
High	2.17	1.59–3.00	< 0.001*	2.80	1.99–3.94	< 0.001**

*Significant level at α = 0.20.

**Significant level at α = 0.05.

The overall prevalence of undiagnosed T2DM was 7.5%: 7.4% in males and 7.5% in females. The greatest prevalence of undiagnosed T2DM was among those aged ≥ 80 years (11.1%), Lisu tribe (10.1%), and farmer (7.8%). Seven factors were found to be associated with undiagnosed T2DM in the univariable logistic regression model: tribe, education, marital status, stress, total cholesterol, LDL-C and triglycerides (Table 2).

However, after adjusting for age and sex, two factors were found to be associated with T2DM in the multivariate model: triglycerides and HDL-C. Those with high triglyceride levels were 2.80 (95% CI 1.99–3.94) times more likely to have T2DM than those with a normal triglyceride level. Those with low HDL-C levels were 1.65 (95% CI 1.16–2.34) times more likely to have T2DM than those with normal HDL-C (Table 2).

## Discussion

The hill tribe adult population aged 30 years and over is characterized by low education levels and poor family economic conditions. More than half are not proficient in Thai, and their linguistic challenges are most pronounced in reading in Thai. Moreover, a quarter of the population have no health insurance, and very few people receive regular preventive healthcare. A large proportion are overweight, and few exercise regularly. They are facing a great challenge with the high proportion of undiagnosed T2DM. The prevalence of undiagnosed T2DM among hill tribe adult individuals aged 30 years and over in Thailand was 7.5%. Triglycerides and HDL-C were identified as factors associated with undiagnosed T2DM among hill tribe people aged 30 years and over in Thailand.

Our study found that the overall prevalence of undiagnosed T2DM among hill tribe adults aged 30 years and over was 7.5%. Mahikul et al. reported that 18.2% of T2DM patients in Thailand were not properly diagnosed^20^. Even though the proportion of undiagnosed T2DM among the hill tribe population is less than that among the entire Thai population, they are living with conditions that contribute to T2DM, such as limited ability to understand Thai, which is the main language used in communicating health messages in Thailand. A large proportion of hill tribes aged 30 years and over work in farming, which is defined as physically demanding work, but many people are categorized as overweight. This might be due to the combined effects of their culinary tradition that favor fatty food^25^ and their lifestyle with little exercise. In addition, 187 participants (7.3%) were excluded from the study due to medical evidence that they had already been treated for T2DM. Therefore, the total prevalence of DM among the study population was 14.3% (264 out of 2552 people).

Although a large proportion of the hill tribe aged 30 years and over were covered by health insurance, few regularly received preventive healthcare, and they were not tested for diabetes. This could be due to poor education, insufficient knowledge and negative opinions towards diabetes prevention. In addition, a large proportion of the hill tribe people are living in poor economic status with family debt. These challenges are further complicated by their limited command of Thai, which might interfere with access to healthcare services. This was supported by a study in Thailand that showed that even when diabetes care had been provided at the community level, the number of clients who accessed the service did not increase significantly^26^.

Some tribes were at a greater risk of developing T2DM than others in the univariable analysis. Being Lisu was associated with greater odds of suffering from T2DM than being Akha in univariable analysis. This is consistent with a study conducted by Tamornpark et al.^27^, which reported that older Lahu, Yao, Karen, and Lisu people were more likely to suffer from T2DM than older Akha people. A literature review identified risk factors for T2DM in different tribes in India, and some tribes were found to be at a significantly greater risk of suffering from T2DM than other tribes^28^. This might be related to genetic, culinary tradition and lifestyle differences among the tribes. Some hill tribes in Thailand prefer to work in the field, while others favor trading, which leads to differences in physical activity and to different rates of T2DM development.

Other socioeconomic factors were found to be associated with T2DM in the univariable analysis, such as education, marital status, and stress. Although these factors were not found to be associated with T2DM in the multivariable analysis, these characteristics might reflect the potential risk of developing T2DM in the future among the hill tribe population. Poor education, marital status and stress were preliminarily associated with T2DM among hill tribe adults. This was supported by a national survey in Thailand, which reported that many socioeconomic statuses were associated with T2DM^29^. There should be a more detailed study to test the causal effect between these variables.

Individuals with higher triglyceride levels were more likely than those with a normal triglyceride level to suffer from T2DM in the hill tribe adult population in Thailand. This is supported by a study conducted in Italy that found that people with high triglyceride levels were significantly more likely to suffer from T2DM than those with low triglyceride levels^30^. Another study found that the association between the level of triglycerides and T2DM was much more significant in males than in females^31^. A study in Thailand confirmed that triglycerides were a major factor contributing to T2DM^29^.

Hill tribe members aged 30 years and over with low levels of HDL-C were more likely to suffer from undiagnosed T2DM. A study in rural Bangladesh reported that low HDL-C was associated with T2DM^32^. A study in Thailand reported that a low level of HDL-C was one significant factor associated with T2DM in the Thai population^33^. This could reflect the impact of poor health behaviors among the hill tribes on T2DM; a large proportion did not exercise (61.0%) and were overweight (60.1%).

Since this is a cross-sectional study, it was not designed to test the causal relationships among the variables. Rather, it is a snapshot assessment aimed at estimating the prevalence of health problems and performing a preliminary examination of the associations between variables in this population by concurrently evaluating both independent variable(s) or exposure(s) and dependent variable(s) or outcome(s). Thus, it is very difficult to interpret the findings in terms of “cause” and “effect”. Therefore, a limitation of this study is that it does not provide any conclusions about the causal relations between variables. Moreover, many variables and subcategories were detected in only a few cases, which could lead to difficulties in the analysis and result in lower statistical power to detect an association. We found that some of the participants, especially those aged over 55 years, had problems communicating in Thai. To address this issue, village public health volunteers who were fluent in both Thai and their (hill tribe) languages were asked to help them complete the questionnaire. Finally, four people did not properly follow the requirement to abstain from food or drink for at least 8 h before the blood specimens were collected; however, their lipid profiles were not different from normal lipid profiles.

## Conclusion

The hill tribe adult population aged 30 years and over in Thailand lives in an environment characterized by low education levels and economic deprivation. Today, the undiagnosed T2DM prevalence among hill tribe people aged 30 years and over is high. A large proportion of hill tribe members have limited knowledge of diabetes prevention and control and negative opinions about DM prevention and control. High triglyceride and low HDL-C levels are factors associated with T2DM among undiagnosed individuals living in these communities. Hill tribe adults should be encouraged to receive regular preventive healthcare. A specific training program needs to be introduced to improve individuals’ knowledge and opinion towards diabetes prevention and control. Finally, an appropriate training program is needed to improve cooking skills and dietary behavior focused on the use of fat for everyday cooking.

## Supplementary Information

Supplementary Table S1.

## Abbreviations

BMI: Body mass index
CI: Confident interval
HbA1c: Glycated hemoglobin
HDL-C: High-density lipoprotein
IOC: Item-objective congruence
IQR: Interquartile range
LDL-C: Low density lipoprotein cholesterol
NCDs: Noncommunicable diseases
SD: Standard deviation
ST-5: Stress Test-5
T2DM: Type 2 diabetes
WHO: World Health Organization
